# Non-tonal L2 proficiency facilitates the perception of tone and pitch contrasts in a tonal L1: evidence from Mandarin–English bilinguals

**DOI:** 10.3389/fpsyg.2026.1746921

**Published:** 2026-04-01

**Authors:** Rong Zhao, Mengjiao Li, Honghao Ren, Hang Wei

**Affiliations:** School of Foreign Studies, Xi’an Jiaotong University, Xi’an, China

**Keywords:** L2 proficiency, Mandarin–English bilinguals, multi-competence theory, pitch perception, tone perception

## Abstract

This study investigated how proficiency in a non-tonal L2 influences the perception of tone-related information in a tonal L1 among Mandarin–English bilinguals who are dominant in Mandarin. In Experiment 1, 65 participants with varying L2 proficiency completed a Mandarin tone perception task involving acoustically similar (T2-T3) and dissimilar (T1-T3 and T2-T4) tone pairs. In Experiment 2, the participants judged pitch height changes in T1, T2, T3, and T4. Results showed that bilinguals with higher L2 proficiency were more sensitive to the T2-T4 contrast and pitch height variations in T1, T2, and T3 than those with lower L2 proficiency. These findings suggest that increased proficiency in a non-tonal L2 selectively and positively influences the perception of tone pairs and pitch contrasts in a tonal L1, and that the effect may occur for the phonological/acoustic features common to English and Mandarin, extending the multi-competence theory to tone-related processing in bilinguals.

## 1 Introduction

Tonal languages are widespread, constituting about 40% of the world’s languages (Maddieson, 2013). Bilingualism is also becoming increasingly common; according to Grosjean (2022), more than half of the world’s population is bilingual. A growing body of research has examined the influence of second language (L2) experience on first language (L1) processing (Dmitrieva, 2019; Takahashi, 2023). Multi-competence theory seeks to elucidate the effects of L2 on L1 and defines multi-competence as “knowledge of two or more languages in one mind” (Cook, 2003). According to this theory (Cook, 2003; Cook and Wei, 2016), the influence of L2 on L1 can be categorized into three types: positive (i.e., L2 experience enhances L1 processing), negative (i.e., L2 experience impairs L1 processing), and neutral (i.e., L2 experience affects L1 processing without significant improvement or deterioration). The theory further proposes that the benefits of learning and using L2 for L1 are numerous. For example, bilinguals can use more complex sentences and read better in their L1. However, if the bilingual’s L1 is no longer dominant (e.g., immigrants), the effects of L2 experience on L1 could be harmful and may lead to L1 attrition (Cook, 2003).

It is noteworthy that the multi-competence theory and previous empirical research have focused primarily on bilinguals of non-tonal languages (e.g., Russian–English bilinguals). For tonal language speakers, the effects of L2 experience on L1 processing, particularly tone processing, remain largely unknown. One study has shown that L2 experience positively influences L1 speech perception among Mandarin–English bilinguals living in mainland China, for whom Mandarin is the dominant language (Zhao et al., 2023). In contrast, another study found L1 tone attrition among Mandarin–English bilinguals dominant in English (Quam and Creel, 2017). However, for L1-dominant Mandarin–English bilinguals, the effects of L2 experience on L1 tone processing remain to be explored. The current study therefore examined this population to investigate how the perception of tone contrasts and pitch height variations in L1 Mandarin might be influenced by L2 English experience. On the basis of prior work (e.g., Zhao et al., 2023; Zinszer et al., 2015; to be detailed below), we hypothesize that positive effects of L2 experience on L1 would occur in phonological/acoustic features that are common to Mandarin and English.

### 1.1 Influence of L2 experience on L1 speech and tone processing in mandarin–English bilinguals

As a tonal language, Mandarin Chinese has drawn considerable attention in bilingualism research. Overall, studies on the influence of L2 experience on L1 speech processing have shown positive effects in Mandarin–English bilinguals (e.g., Li et al., 2016; Zhao et al., 2023). L2 experience is a broad term and has been operationalized as L2 proficiency, length of residence in the target country, L2 age of acquisition (AoA), and so on (e.g., Li et al., 2016; Zhao et al., 2023; Zinszer et al., 2015). Zhao et al. (2023) tested Mandarin–English bilinguals with varying levels of L2 proficiency as measured by the Oxford Quick Placement Test (OQPT). They found that participants’ perceptual sensitivity to L1 speech (consonant, vowel and tone deviants) increased with L2 proficiency in a quiet environment. Li et al. (2016) reported similar findings under noisy backgrounds. Their study involved two groups of participants: Chinese native listeners who had resided in the US (CNU) for 1–3 years, and Chinese native listeners living in China (CNC) with no history of residence in English-speaking countries. Participants were asked to identify Mandarin vowel-plus-tone in quiet, stationary and temporally-modulated noise, babble-modulated noise, and multi-talker babble. The results showed that CNU listeners achieved significantly higher identification scores than CNC listeners under most noisy conditions. These findings suggest that L2 English experience may enhance L1 Mandarin speech processing in Mandarin–English bilinguals.

However, the effects of L2 experience on L1 tone processing are not yet fully understood. One study reported a negative effect: Mandarin–English bilinguals dominant in L2 showed tone attrition in L1 word recognition (Quam and Creel, 2017). The participants arrived in the US during childhood. Most had learned English after Mandarin and were therefore considered L2 English speakers. Participants were shown two pictures (whose names differed in tone, vowel, or both) and were asked to choose the one matching the final word of the heard sentence. Results showed that bilinguals with more L2 experience had slower reaction times and lower accuracy in tone-disambiguated trials. The authors concluded that more exposure to English and less exposure to Mandarin may lead to tone attrition in word recognition among Mandarin–English bilinguals (Quam and Creel, 2017).

Nevertheless, for L1-dominant Mandarin–English bilinguals, the influence of L2 experience on L1 tone processing remains largely unclear. To the best of our knowledge, Zinszer et al. (2015) is the only study that has investigated this issue. Specifically, they used functional near-infrared spectroscopy to examine the influence of L2 AoA on categorical perception of lexical tones in L1. The participants were L1-dominant Mandarin–English bilinguals from a local university in China. The results showed that perceptual sensitivity of the left middle temporal gyrus to tonal categories is modulated by L2 AoA. However, Zinszer et al. (2015) observed this effect only in a single brain region but did not find any relationship between L1 tone processing and L2 AoA in behavioral measures. Thus, additional studies are needed to clarify how L2 experience affects L1 tone processing in L1-dominant Mandarin–English bilinguals. Given that the available evidence is very limited, below we review studies comparing sensitivity to Mandarin tone-related information between native English and Chinese listeners to provide preliminary evidence on the potential effects of non-tonal L2 experience on L1 tone processing. This review could help shed light on shared and non-salient phonological/acoustic features in Chinese and English.

### 1.2 Differences in sensitivity to mandarin tones and pitch cues between native English and Chinese listeners

Mandarin has four lexical tones (plus a neutral tone in specific grammatical contexts): high level (T1, e.g., /ma1/ “mother”), high rising (T2, e.g., /ma2/ “hemp”), low falling-rising (T3, e.g., /ma3/ “horse”), and high falling (T4, e.g., /ma4/ “scold”). These tones are distinguished by both pitch height and pitch contour (Jongman et al., 2017; Jongman et al., 2006). Acoustically, T2-T3 pair is more similar than T1-T3 or T2-T4 pair because the pitch contours of T2 and T3 both start with a dip followed by a rise (So and Best, 2010). Previous studies have compared perceptual sensitivity to acoustically similar (e.g., T2-T3) and dissimilar tone pairs (e.g., T1-T3) between native English and Chinese listeners (Chandrasekaran et al., 2007; So and Best, 2010; Yang et al., 2020), as well as sensitivity to pitch cues (e.g., pitch height vs. pitch slope) of Mandarin tones (Jongman et al., 2017; Liu, 2013).

One group of studies has shown that sensitivity to Mandarin tone pairs/tones differs between native English and Chinese listeners (Chandrasekaran et al., 2007; Jongman et al., 2017; So and Best, 2010). Compared with native English listeners, native Mandarin listeners showed greater sensitivity to the T1-T3 contrast (acoustically dissimilar) at the pre-attentive stage, as evidenced by a stronger mismatch negativity in an ERP study using the oddball paradigm. In contrast, no significant group difference was found for the T2-T3 contrast (acoustically similar) (Chandrasekaran et al., 2007). Another study reported that native Mandarin listeners were more sensitive to T4 variations, as indicated by lower just noticeable differences, whereas native English listeners were more sensitive to T2 variations (Jongman et al., 2017). Similarly, So and Best (2010) found that native English listeners were less sensitive to T4 than Cantonese (another tonal language in China) and Japanese listeners, as reflected in lower tonal identification scores. Taken together, these studies show that native Chinese and English listeners differ in their sensitivity to Mandarin tone contrasts/tones.

Building on these findings, an intriguing question arises: Does increased experience with L2 English affect Mandarin–English bilinguals’ sensitivity to Mandarin tone contrasts (specifically T1-T3, T2-T3, and T2-T4)? If so, does this effect vary across tone pairs (e.g., positive or negative effect)?

Another group of studies has found differences in sensitivity to pitch cues in Mandarin tones between native English and Mandarin listeners (Jongman et al., 2017; Liu, 2013). Specifically, native English listeners are more sensitive to pitch height, whereas native Mandarin listeners are more sensitive to pitch slope. For example, Jongman et al. (2017) reported that native English listeners showed lower just noticeable differences (indicating stronger sensitivity) for pitch height, while native Mandarin listeners showed lower just noticeable differences for pitch slope. A similar pattern was observed when comparing native English and Thai listeners (Gandour and Harshman, 1978).

In addition, pitch height has been regarded as the most salient pitch characteristic for distinguishing tones. Gandour and Harshman (1978) demonstrated this by selecting 13 tones and encompassing five pitch characteristics (height, direction, range, slope, and beginning and ending pitch points) commonly used to distinguish tones across tonal languages. They asked native listeners of Thai, Yoruba, and English to rate the dissimilarity of pitch patterns between tone pairs using an 11-point scale, and found that pitch height was more salient than the other four pitch characteristics for all three groups.

These findings on pitch height provide an additional perspective on the effects of L2 experience on L1 tone perception. Whether and how increased experience with L2 English influences the perception of pitch height changes in the four Mandarin tones among L1-dominant Mandarin–English bilinguals remains to be explored.

### 1.3 The present study

The current study aimed to examine the influence of L2 experience on L1 tone perception in L1-dominant Mandarin–English bilinguals, focusing on both tone pairs and pitch height changes. Most participants in this study began formal English learning in childhood (see Table 1 for details). Moreover, they are required to complete mandatory English courses and examinations from elementary school through university. L2 experience was operationalized as L2 proficiency, since previous research has identified L2 proficiency as a key factor influencing L1 speech processing (Yang and Fox, 2017; Zhao et al., 2023).

**Table 1:** The participants’ language background information in this study.

	L1 (Mandarin)			L2 (English)		
	Mean	SD	Range	Mean	SD	Range
L2 AoA (years)				8.18	2.38	4–14
Listening	5.97	0.88	3–7	3.92	1.24	1–6
Speaking	5.80	0.89	3–7	3.51	1.25	1–6
Reading	6.11	0.71	5–7	4.71	1.21	2–6
Writing	5.54	1.06	2–7	4.17	1.07	2–7
OQPT-total				39.40	7.52	20–54
Exp1-lower L2				33.09	4.65	20–39
Exp1-higher L2				45.38	3.53	40–54
Exp2-lower L2				33.09	4.65	20–39
Exp2-higher L2				45.59	3.60	40–54

L2 AoA, L2 age of acquisition.

Lines 2 to 5 present participants’ self-rated proficiency in listening, speaking, reading, and writing in both their L1 and L2, using a 7-point scale, where higher scores indicate better language skills.

The final five lines report the Oxford Quick Placement Test (OQPT) scores for all participants, as well as for the lower and higher L2 proficiency groups in Experiment 1 and Experiment 2, respectively. Higher OQPT scores reflect higher L2 proficiency. The OQPT scores of the two groups in Experiment 1 and Experiment 2 were slightly different because the outlier exclusions led to slightly different subsets of participants in each experiment.

Experiment 1 investigated how L2 proficiency may influence perception of similar (T2-T3) and dissimilar (T1-T3 and T2-T4) tone pairs in L1. On the one hand, previous research has shown that L2 AoA affects neural sensitivity to the categorical perception of the T2-T4 contrast (Zinszer et al., 2015). On the other hand, English has a rising-falling intonation contrast that resembles the Mandarin T2-T4 contrast (for example, a single word such as “yes” may carry a rising pitch to indicate a question or a falling pitch to indicate a statement). Based on these considerations, we predicted that Mandarin–English bilinguals would become more sensitive to the T2-T4 contrast with increasing L2 experience.

Experiment 2 examined how L2 proficiency may influence perception of pitch height changes in the four L1 tones (T1, T2, T3, and T4). Given that pitch height is also a primary acoustic cue in English phonology (Chrabaszcz et al., 2014; Gussenhoven, 2004), underpinning both lexical stress and sentence-level intonation, and that native English listeners are more sensitive to pitch height than native Mandarin listeners (Jongman et al., 2017), we predicted that the L2 experience of Mandarin–English bilinguals would positively influence their perception of pitch height changes in Mandarin tones, with the exception of T4. This exception is motivated by previous research showing that native English listeners are less sensitive to T4 variations than native Mandarin listeners (Jongman et al., 2017). This suggests that lexical-level phonological units such as T4 may not be salient in English, and that native Mandarin listeners may not develop enhanced perceptual sensitivity to T4 variations during English learning.

## 2 Experiment 1: perception of tone pairs

To investigate the effect of non-tonal L2 proficiency on L1 tone perception, Experiment 1 examined how L1-dominant Mandarin-English bilinguals with different L2 proficiency levels discriminate acoustically similar and dissimilar tone pairs in L1.

### 2.1 Method

#### 2.1.1 Participants

Sixty-five native Mandarin speakers^1^ (33 males, mean age = 19.97 years, *SD* = 1.89) participated in this study. All were students at Xi’an Jiaotong University (located in central China) and had learned English as their L2. Their language background information is provided in the *Measures* section and in Table 1. All participants reported good mental and physical health, normal hearing, and no formal music training. None of them had lived in an English-speaking country for more than 3 months. All participants provided informed consent and were paid for their participation.

#### 2.1.2 Measures

Participants’ language proficiency was measured using a simple self-assessment questionnaire and the Oxford Quick Placement Test (OQPT). In the self-assessment questionnaire, they rated their proficiency in listening, speaking, reading and writing in both L1 and L2 on a seven-point scale from very poor (1) to excellent (7). They were also asked to write down the age at which they began learning English (L2 age of acquisition, L2 AoA).^2^ The OQPT was used to assess general L2 proficiency and has been shown to be a reliable measure for Chinese–English bilinguals (Zhang et al., 2020). It has also been widely used in previous speech processing studies (Wang and Treffers-Daller, 2017; Zhao et al., 2023). The test consists of 60 single-choice questions (one point each). The paper and pen version of the OQPT was used in the current study and takes about 30 min to complete.

Participants’ language background information is summarized in Table 1. Paired sample t-tests showed that participants’ proficiency in listening (*t* (64) = 14.16, *p* < 0.001), speaking (*t* (64) = 14.68, *p* < 0.001), reading (*t* (64) = 9.65, *p* < 0.001) and writing (*t* (64) = 9.69, *p* < 0.001) was significantly higher in L1 than in L2. In addition, participants used Mandarin, their native language, for most academic activities, while English was used only in a limited subset of academic tasks. Mandarin was also the language used in both family and social contexts. According to the criteria established in previous research (Lim et al., 2008; Treffers-Daller, 2019), they were L1-dominant bilinguals. Participants’ OQPT scores ranged from 20 to 54^3^, indicating that their L2 proficiency levels ranged from elementary to advanced (Geranpayeh, 2003). Specifically, 6 participants were at the elementary level (18–29 points), 26 at the lower intermediate level (30–39), 24 at the upper intermediate level (40–47), and 9 at the advanced level (48–54). For subsequent analyses, participants were divided into two groups based on their OQPT scores to examine how L2 proficiency influences L1 processing (for details, see the *Reaction time analysis* section).

#### 2.1.3 Materials

**Figure 1:**
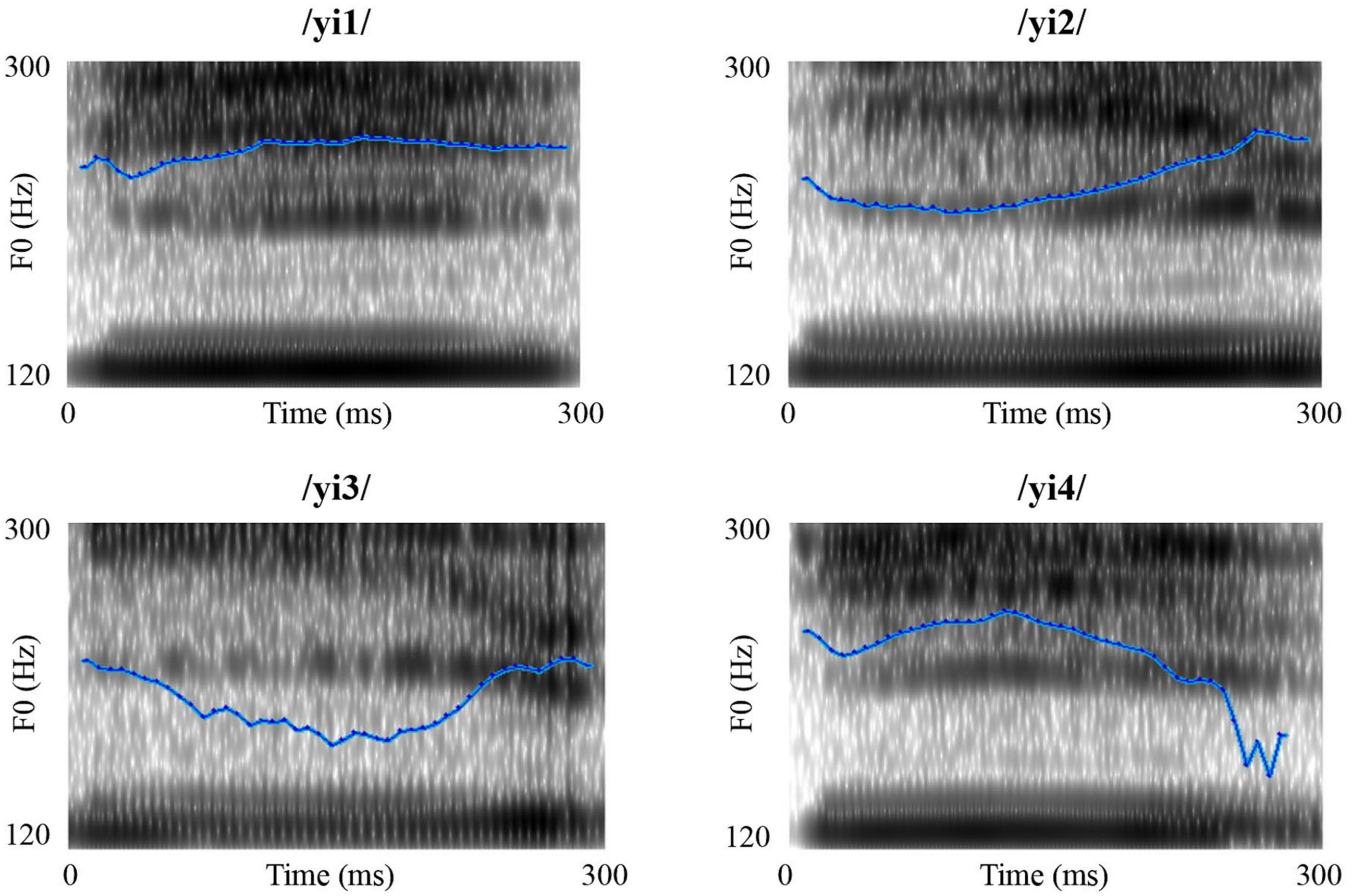
The spectrograms of the four Mandarin syllables used in experiment 1.

The materials for this experiment were four Mandarin monosyllables (see Figure 1): yi1 (T1), yi2 (T2), yi3 (T3), and yi4 (T4). These syllables share identical segmental information but differ in tone, allowing us to avoid confounding effects from non-tonal cues. They are common in Mandarin and have been widely used in previous studies on speech perception (Lee et al., 2012; Politzer-Ahles et al., 2016). Based on previous studies (Chandrasekaran et al., 2007; Jongman et al., 2017; So and Best, 2010), T2 and T3 formed an acoustically similar tone pair, whereas T1 and T3, and T2 and T4 formed acoustically dissimilar tone pairs. Each syllable was pronounced more than 20 times by a female native Mandarin speaker. The clearest version of each syllable was evaluated and selected by three native Mandarin speakers for use in the experiment. All selected syllables were normalized to a duration of 300 ms^4^ and an intensity of 70 dB using Praat software.^5^

#### 2.1.4 Procedure

The syllables were presented binaurally through headphones (HD 280 PRO, Sennheiser) in a quiet laboratory using an active oddball paradigm (Rong et al., 2023; Zhao et al., 2023). The experiment consisted of three sessions, each session containing two blocks. Each tone pair was counterbalanced across the two blocks, with one tone serving as the standard stimulus while the other serving as the deviant stimulus in one block, and vice versa in the other block (Gu et al., 2013; Tang et al., 2021). For example, for the T1-T3 contrast, T1 served as the standard and T3 as the deviant in block 1, while T1 served as the deviant and T3 as the standard in block 2. The presentation order of the three sessions was counterbalanced across participants.

Each block contained 300 standard and 50 deviant syllables presented pseudo-randomly. All blocks began with 10 standard syllables to help participants adapt to the experiment. There were at least 3 standard syllables between any two deviant syllables. The interval between syllables was 650 ms. Participants were instructed to listen to a sequence of syllables and press the “space” key as quickly and accurately as possible upon hearing a different sound. Stimulus presentation and data collection (reaction time and accuracy) were conducted using E-Prime 2.0 (Schneider et al., 2012). There was a practice session before the main experiment to familiarize participants with the task. Participants could take a break after each session. The entire experiment lasted about 40 min.

#### 2.1.5 Data analysis

Participants’ reaction time (RT) and accuracy (ACC) data were treated as dependent variables. Data from one participant were excluded because his/her error rate (0.40) was 2.5 SD (0.05) above the overall mean error rate (0.03) (Darcy and Thomas, 2019; Nijveld et al., 2022). The error rates of the remaining participants were all below 0.15. Mixed-effects models were constructed using the lme4 package (Bates et al., 2014) and the lmerTest package (Kuznetsova et al., 2017) in R version 4.4.2. The data, analysis code and results are available at (see text footnote 3).

To examine whether bilinguals with higher and lower L2 proficiency differ significantly in their perceptual sensitivity to different tone pairs, participants were divided into a lower level group (*n* = 32) and a higher level group (*n* = 32) using the median of the OQPT scores (39.5) as the cut-off point (Wang and Treffers-Daller, 2017; Whitford and Titone, 2012). The mean RTs and ACC for the perception of each tone pair in each participant group (see text footnote 3).

Before constructing the linear mixed-effects models, RTs were log-transformed to reduce skewness. Trials in which participants responded incorrectly were deleted and RTs shorter than 150 ms were removed (Ong et al., 2020; Zhao et al., 2023). In line with prior research (Feng et al., 2021; Ong et al., 2020), RTs more than 2.5 SDs from the mean of each condition were removed using the sdTrim function. In total, 3.14% of the data (602 trials) were excluded from further analysis.

The linear mixed-effects models were then built on the RT data, with L2 proficiency (lower level group vs. higher level group), Condition (T2-T3 vs. T1-T3 vs. T2-T4 contrasts) and their interaction as fixed factors. L2 proficiency was contrast coded (lower level group = −0.5, higher level group = 0.5), and Condition was treatment coded (with the T2-T4 contrast as the reference level). Forward model comparisons^6^ were used to determine the best-fitting random effects structure using the anova function (Cunnings, 2012). Specifically, the anova() function compares two models using likelihood ratio tests to assess whether the addition of the random effects parameter significantly improves the model fit (i.e., explains more variance in the data). We employed a stepwise approach to compare models with increasingly complex random effects structures, ultimately identifying the optimal random effects model.

The logistic mixed-effects models were then built on the ACC data. The methods for variable encoding, model building and model comparison were the same as described above.

Subsequently, because the fixed effects estimated by the mixed-effects model represent differences relative to the reference level (T2-T4 contrast), and one independent variable in Experiment 1 had three levels (i.e., Condition: T2-T4, T2-T3, and T1-T3 contrasts), the model output does not directly provide results for all pairwise comparisons at once. Thus, all interaction effects were obtained by changing the reference level, and *post hoc* pairwise comparisons for all fixed factors were conducted using the emmeans function with Tukey adjustments (Lenth et al., 2022). Effect sizes in the linear mixed-effects model were subsequently calculated using the eff_size function from emmeans (Lenth et al., 2022).^7^ For the logistic mixed-effects model, effect sizes were quantified by calculating odds ratios (OR) (Kosaka, 2024). An OR greater than 3 or less than one third indicates that the effect is strong (Haddock et al., 1998).

In addition, mixed-effects models were built with L2 proficiency treated as a continuous variable for both the RT and ACC data, as recommended by one reviewer. The methods for categorical variable encoding, model building, and model comparison were the same as those described above. *Post hoc* pairwise comparisons were conducted for the interaction effects using emtrends() to obtain the effects of interest (Lenth et al., 2022).

### 2.2 Results

#### 2.2.1 Reaction time

**Figure 2:**
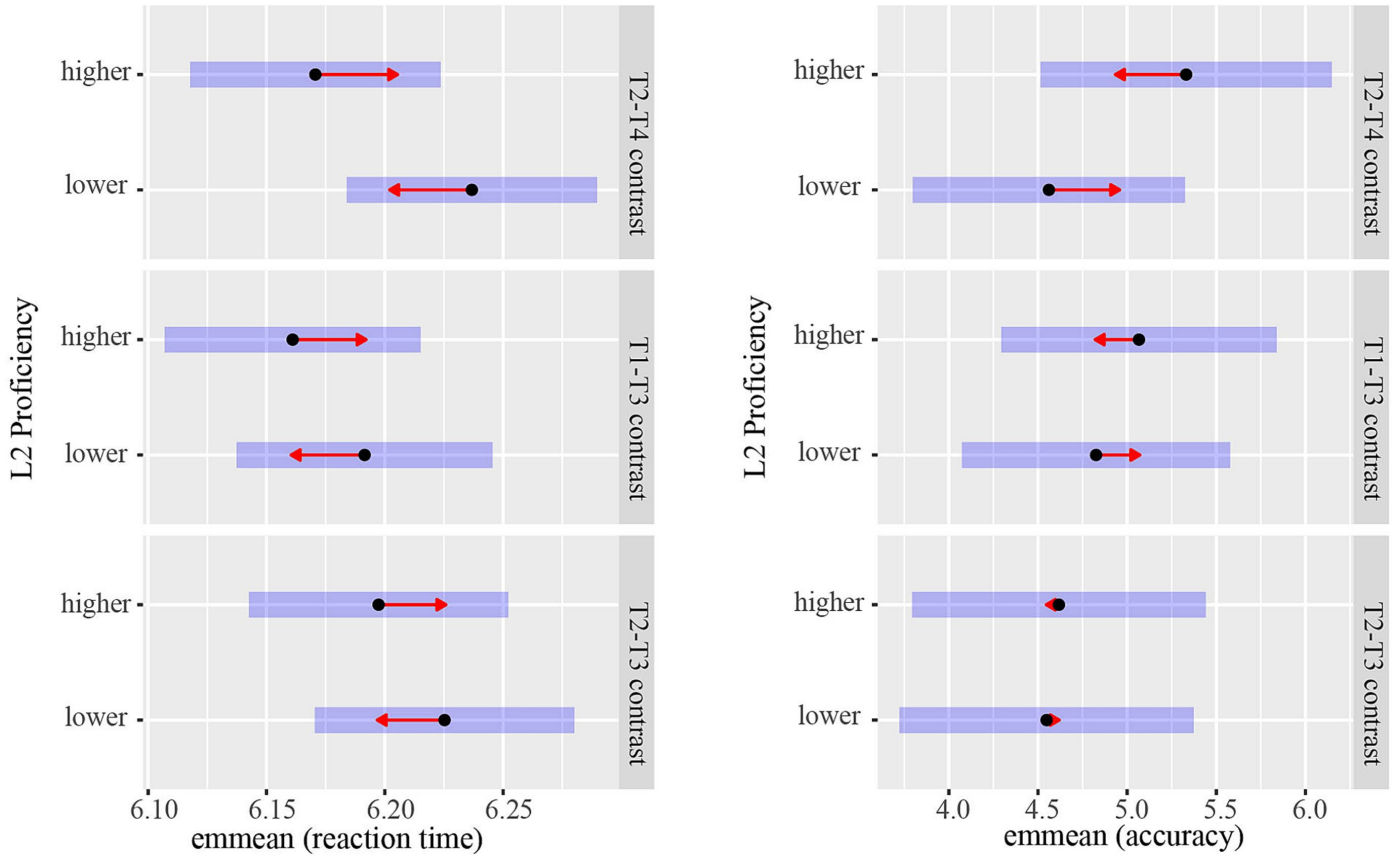
Group differences between bilinguals with higher and lower L2 proficiency in the perception of the T2-T4, T1-T3, and T2-T3 contrasts. The figure displays estimated marginal means for reaction times and accuracy. Pairwise comparisons between proficiency groups were conducted within each contrast. The higher proficiency group perceived the T2-T4 contrast faster and more accurately than the lower proficiency group. The degree of overlap between arrows reflects, as closely as possible, the significance of the comparison between the two estimates.

The results of the best model (with L2 proficiency as a categorical variable; see Table 2) indicated that only the interaction effect between L2 proficiency and Condition was significant (*β* = 0.04, *SE* = 0.02, *t* = 2.31, *p* = 0.024), suggesting that the effect of L2 proficiency on the perception of the T2-T4 contrast was larger than that on the T2-T3 contrast (see Figure 2). The other effects were not statistically significant (*ps* > 0.05).

**Table 2:** Mixed-effects model results for RTs in Experiment 1 with L2 proficiency as a categorical variable.

Fixed effects	*β*	*SE*	*t*	*p*
T2-T4 contrast as the reference level				
Intercept	6.20	0.02	304.22	<0.001***
Condition: T1-T3 contrast	−0.03	0.02	−1.53	0.155
Condition: T2-T3 contrast	<0.01	0.02	0.46	0.657
L2 proficiency	−0.07	0.04	−1.87	0.067
T1-T3 contrast × L2 proficiency	0.04	0.02	1.63	0.109
T2-T3 contrast × L2 proficiency	0.04	0.02	2.31	0.024*
T1-T3 contrast as the reference level				
Intercept	6.18	0.02	297.07	<0.001***
Condition: T2-T3 contrast	0.04	0.02	2.02	0.071
L2 proficiency	−0.03	0.04	−0.84	0.406
T2-T3 contrast × L2 proficiency	<0.01	0.02	0.13	0.898
T2-T3 contrast as the reference level				
Intercept	6.21	0.02	295.03	<0.001***
L2 proficiency	−0.03	0.04	−0.75	0.454

*p < 0.05; ***p < 0.001.

The *post hoc* analysis of Condition showed no significant differences (*ps* > 0.05) in the speed at which participants perceived three pairs of tone contrast (T2-T4 vs. T2-T3 vs. T1-T3). The *post hoc* analysis of L2 proficiency found no significant difference (*p* = 0.229) between the higher and lower level groups. The *post hoc* analysis of the interaction between L2 proficiency and Condition showed that L2 proficiency might affect the perception of T2-T4 contrast (*β* = 0.07, *SE* = 0.04, *z* = 1.87, *p* = 0.062, *d* = 0.40), with the higher level group perceiving T2-T4 contrast numerically faster than the lower level group (see Figure 2 and see text footnote 3). However, the speed of perception of T1-T3 contrast (*β* = 0.03, *SE* = 0.04, *z* = 0.84, *p* = 0.403, *d* = 0.19) and T2-T3 contrast (*β* = 0.03, *SE* = 0.04, *z* = 0.75, *p* = 0.451, *d* = 0.17) was not affected by L2 proficiency.

The results of the best model treating L2 proficiency as a continuous variable (see text footnote 3) are briefly reported, as recommended by the reviewer. The effects of Condition, L2 proficiency, and their interaction were not statistically significant (*ps* > 0.05). *post hoc* analyses of the interaction between Condition and L2 proficiency (see text footnote 3) showed that the simple effects of L2 proficiency on the T2-T4, T1-T3, and T2-T3 contrasts were also not statistically significant (*ps* > 0.05).

#### 2.2.2 Accuracy

The results of the optimal model (with L2 proficiency as a categorical variable; see Table 3) showed that none of the effects reached statistical significance (*ps* > 0.05). Although the interaction between L2 proficiency and Condition was only marginally significant (the effect of L2 proficiency on the T2-T4 contrast vs. the T2-T3 contrast: *β* = −0.70, *SE* = 0.41, *t* = −1.72, *p* = 0.086), we nevertheless conducted *post hoc* pairwise comparisons because it was directly relevant to the aims of the present study.

**Table 3:** Mixed-effects model results for ACC in Experiment 1 with L2 proficiency as a categorical variable.

Fixed effects	*β*	*SE*	*z*	*p*
T2-T4 contrast as the reference level				
Intercept	4.95	0.35	14.15	<0.001***
Condition: T1-T3 contrast	<0.01	0.46	<0.01	1.00
Condition: T2-T3 contrast	−0.36	0.45	−0.81	0.419
L2 proficiency	0.77	0.40	1.90	0.057
T1-T3 contrast×L2 proficiency	−0.53	0.43	−1.23	0.219
T2-T3 contrast×L2 proficiency	−0.70	0.41	−1.72	0.086
T1-T3 contrast as the reference level				
Intercept	4.95	0.34	14.48	<0.001***
Condition: T2-T3 contrast	−0.36	0.46	−0.80	0.427
L2 proficiency	0.24	0.37	0.65	0.517
T2-T3 contrast×L2 proficiency	−0.17	0.44	−0.39	0.694
T2-T3 contrast as the reference level				
Intercept	4.58	0.36	12.88	<0.001***
L2 proficiency	0.07	0.44	0.16	0.876

***p < 0.001.

The *post hoc* analysis of Condition showed no significant differences (*ps* > 0.05) in the accuracy with which participants perceived the three tone pairs. The *post hoc* analysis of L2 proficiency found that participants with higher and lower L2 proficiency perceived tone pairs with similar accuracy (*p* = 0.271). The *post hoc* analysis of the interaction between L2 proficiency and Condition showed that the perception of T2-T4 contrast appeared to be affected by L2 proficiency (*β* = −0.77, *SE* = 0.40, *z* = −1.90, *p* = 0.057, *OR* = 0.46). Participants with higher L2 proficiency perceived the T2-T4 contrast more accurately (numerically) than those with lower L2 proficiency (see Figure 2 and see text footnote 3). However, L2 proficiency did not influence the perception of T1-T3 (*β* = −0.24, *SE* = 0.37, *z* = −0.65, *p* = 0.517, *OR* = 0.79) or T2-T3 contrast (*β* = −0.07, *SE* = 0.45, *z* = −0.16, *p* = 0.876, *OR* = 0.93).

The results of the best model treating L2 proficiency as a continuous variable (see text footnote 3) showed that the effects of Condition and L2 proficiency were not significant (*ps* > 0.05). However, their interaction was significant (i.e., the effect of L2 proficiency on the T2-T4 contrast vs. the T2-T3 contrast, *p* = 0.035). Follow-up analyses of the interaction between L2 proficiency and Condition (see text footnote 3) showed that the simple effects of L2 proficiency on the T2-T4, T1-T3, and T2-T3 contrasts were also not statistically significant (*ps* > 0.05).

### 2.3 Discussion

Experiment 1 investigated how L2 proficiency influenced Mandarin–English bilinguals’ perception of acoustically similar (T2-T3) and dissimilar (T2-T4, T1-T3) tone contrasts in L1. The results of the best model treating L2 proficiency as a categorical variable showed that the interaction effect between L2 proficiency and Condition was significant in the RT data and marginally significant in the ACC data, suggesting that the difference in performance between participants with higher and lower L2 proficiency in perceiving the T2-T4 contrast differed from that in perceiving the T2-T3 contrast. Subsequent analyses suggest that perception of the T2-T4 contrast appeared to be influenced by bilinguals’ L2 proficiency: bilinguals with higher L2 proficiency perceived the T2-T4 contrast numerically faster and more accurately than those with lower L2 proficiency. However, perception of the T1-T3 and T2-T3 contrasts was not influenced by L2 proficiency. Taken together, these findings suggest a possible positive and selective effect of non-tonal L2 proficiency on the perception of L1 tone pairs.

The results of the best model treating L2 proficiency as a continuous variable showed that some of the effects of interest in this study became non-significant, which may have been due to the limited number of participants. We conducted power simulations (for details, see text footnote 3) based on the current data and models (with L2 proficiency treated as a continuous variable). The results indicated that, as the number of participants increased, the pattern of results was consistent with that reported in this paper when L2 proficiency was treated as a categorical variable. Therefore, in the following General Discussion, we primarily focused on the results in which L2 proficiency was treated as a categorical variable.

Tones are distinguished by pitch variations, yet it remains unclear whether L2 proficiency also positively influences the perception of pitch cues. Given that pitch height is an important acoustic cue in both English phonology (lexical stress and sentence-level intonation) and Mandarin tones (Chrabaszcz et al., 2014; Gussenhoven, 2004; Jongman et al., 2017), and that native English listeners are more sensitive to pitch height than native Mandarin listeners (Jongman et al., 2017), Experiment 2 examined the extent to which L2 proficiency influences Mandarin–English bilinguals’ perception of pitch height variations in L1 tones.

## 3 Experiment 2: perception of pitch height contrasts

To explore the potential impact of non-tonal L2 proficiency on the perception of pitch cues in a tonal L1, Experiment 2 investigated how Mandarin–English bilinguals with varying L2 proficiency levels discriminate pitch height variations in L1 tones (T1, T2, T3, and T4).

### 3.1 Method

#### 3.1.1 Participants

The participants were the same as in Experiment 1. All participants first completed Experiment 1, followed by the OQPT, and then completed Experiment 2.

#### 3.1.2 Materials

**Figure 3:**
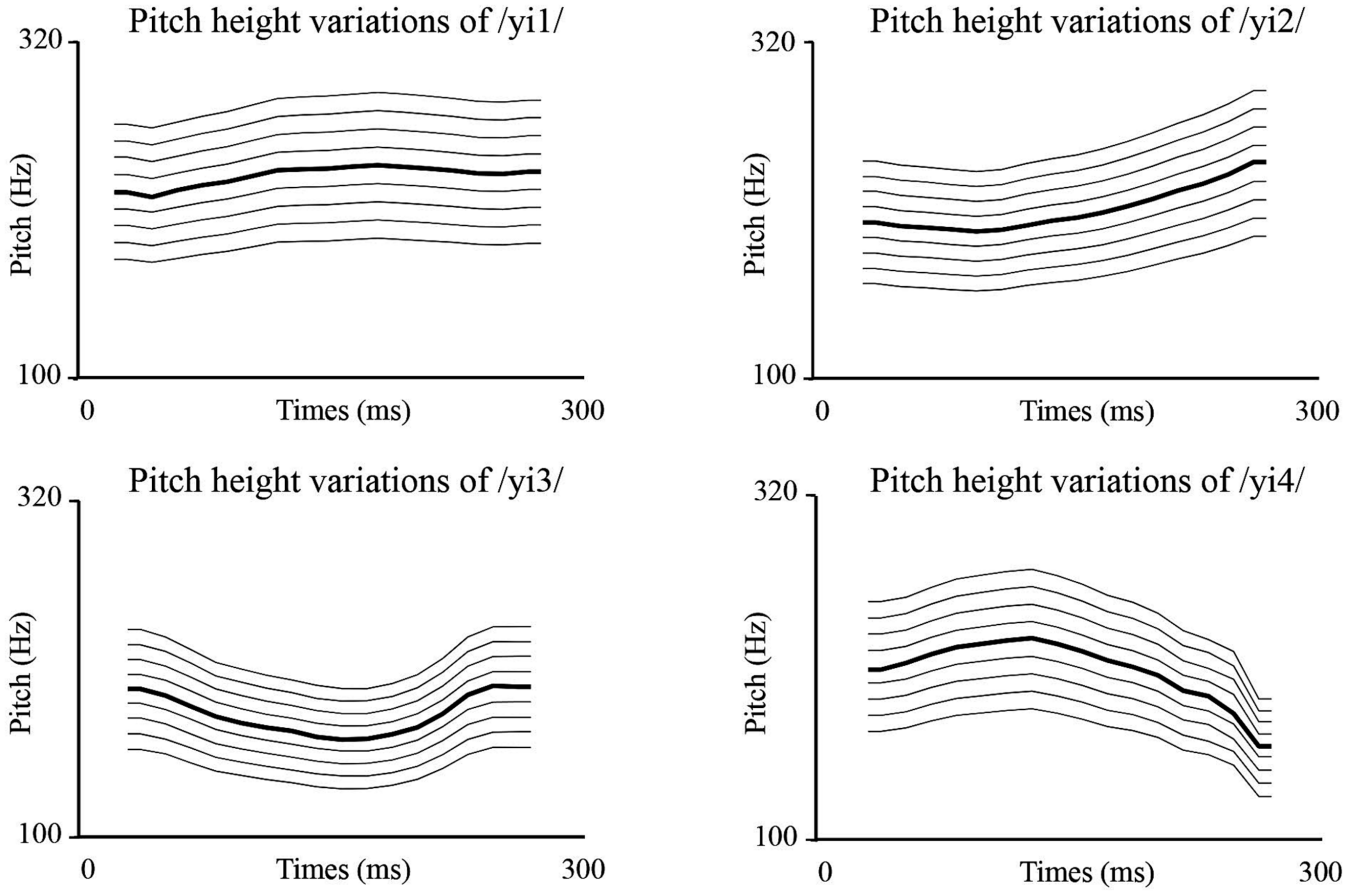
The pitch patterns of the stimuli used in experiment 2. The bold line represents the pitch of the original syllable. The non-bold pitch lines, from top to bottom, represent the original F0 increased by 20, 15, 10, and 5%, and decreased by 5, 10, 15, and 20%.

The materials for Experiment 2 were created by changing the overall F0 of the four syllables from Experiment 1. Eight new versions of each syllable were created by decreasing or increasing the F0 by 5, 10, 15, and 20% (Barcroft and Sommers, 2014). For instance, if an unmodified syllable had an F0 of 200 Hz at a given time point, the F0 would be changed to 190 Hz for the 5% decrease stimulus and 210 Hz for the 5% increase stimulus. These eight new stimuli were created using Praat software^8^ (see text footnote 5). Each syllable therefore had nine versions in total: the original stimulus, stimuli with 5, 10, 15, and 20% F0 decreases, and stimuli with 5, 10, 15, and 20% F0 increases (see Figure 3). Prior to the experiment, three native Mandarin speakers evaluated the naturalness of the resynthesized stimuli, and all confirmed that the stimuli sounded natural.

#### 3.1.3 Procedure

Experiment 2 consisted of four blocks, each containing only one type of syllable/tone. Each block included 72 stimulus pairs formed by the nine versions of each syllable. The two stimuli in each pair differed only in pitch. There were 8, 7, 6, 5, 4, 3, 2, and 1 pairs corresponding to pitch increases or decreases of 5, 10, 15, 20, 25, 30, 35, and 40%, respectively. We also constructed 36 additional pairs in which one of the nine versions was paired with itself (each pair appeared four times). Thus, each block comprised 72 different stimulus pairs and 36 identical pairs. The 2:1 ratio of different to identical pairs followed previous studies (e.g., 3:1 in Huang and Johnson, 2010; 10:7 in Xu et al., 2006). The 108 stimulus pairs were presented randomly within a block. The order of presentation of the four blocks was counterbalanced across participants.

At the beginning of each block, a “+” appeared on the screen and blinked three times to signal the upcoming speech stimuli. The stimuli were then presented in pairs with an interstimulus interval of 500 ms. Participants were instructed to judge as quickly and accurately as possible whether the pitch of the two stimuli was the same or different. For identical stimuli, participants were instructed to press the “F” key with their left hand; for different stimuli, they were instructed to press the “J” key with their right hand. The key assignments were counterbalanced across participants. The maximum response time was 3,000 ms, followed by a 1,000 ms blank after the keypress before the next trial. Before the experiment, participants were presented with a practice session to familiarize themselves with the task. They could take a rest after each block. Stimulus presentation and data collection were performed using E-Prime 2.0 (Schneider et al., 2012). The entire experiment lasted about 25 min.

#### 3.1.4 Data analysis

One participant’s data was removed because his/her error rate (0.54) was 2.5 SD (0.08) beyond the overall mean error rate (0.32) (Darcy and Thomas, 2019; Nijveld et al., 2022). Note that the mean error rate was higher in Experiment 2 than in Experiment 1. This is probably due to the greater difficulty of the task used in Experiment 2, with the pitch difference between two tones ranging from 7.8 to 97.2 Hz, which is highly demanding to perceive. As in Experiment 1, mixed-effects models were constructed for RT and ACC data using the lme4 package (Bates et al., 2014) and the lmerTest package (Kuznetsova et al., 2017) in R version 4.4.2. The data, analysis code and results are available (see text footnote 3).

To examine whether bilinguals with higher and lower L2 proficiency show different sensitivities to pitch height variations in tones, participants were divided into lower (*n* = 32) and higher level groups (*n* = 32) based on the median of their OQPT scores (39.5) (Wang and Treffers-Daller, 2017; Whitford and Titone, 2012). The mean RTs and ACC for perceiving the pitch changes of each tone in each group of participants are summarized (see text footnote 3).

Similar data trimming procedure as that used in Experiment 1 was adopted here, which excluded 33.20% of the data (9,178 trials) from further analysis. The high proportion of data exclusion results from the application of the exclusion criteria to Experiment 2, in which a relatively high mean error rate (0.32) led to a larger number of trials being excluded.

Next, linear mixed-effects models were constructed on the RT data, with L2 proficiency (lower level group vs. higher level group), Condition (T1 vs. T2 vs. T3 vs. T4), and their interaction as fixed factors. L2 proficiency was contrast coded (lower level group = −0.5, higher level group = 0.5), and Condition was treatment coded (with T1 as the reference level). The method for determining the best-fitting random effects structure was the same as Experiment 1.

The logistic mixed-effects models were then constructed for the ACC data. The methods for variable coding, model building and model comparison were the same as those for the RT data.

Subsequently, because the fixed effects estimated by the mixed-effects model represent differences relative to the reference level (T1 pitch height changes), and because one independent variable in Experiment 2 had four levels (i.e., Condition: T1, T2, T3, and T4 pitch height changes), the model output does not directly provide all pairwise comparisons at once. Therefore, all interaction effects were examined by changing the reference level, and *post hoc* pairwise comparisons for all fixed factors were conducted using the emmeans function with Tukey adjustments (Lenth et al., 2022). Effect sizes were calculated using the same methods as in Experiment 1.

Similar to Experiment 1, mixed-effects models were constructed for both the RT and ACC data, with L2 proficiency entered as a continuous predictor, as recommended by one of the reviewers. The methods for coding categorical variables, model building, and model comparison were identical to those described above. *post hoc* pairwise comparisons for the interaction effects were conducted using emtrends() to estimate the effects of interest (Lenth et al., 2022).

### 3.2 Results

#### 3.2.1 Reaction time

**Figure 4:**
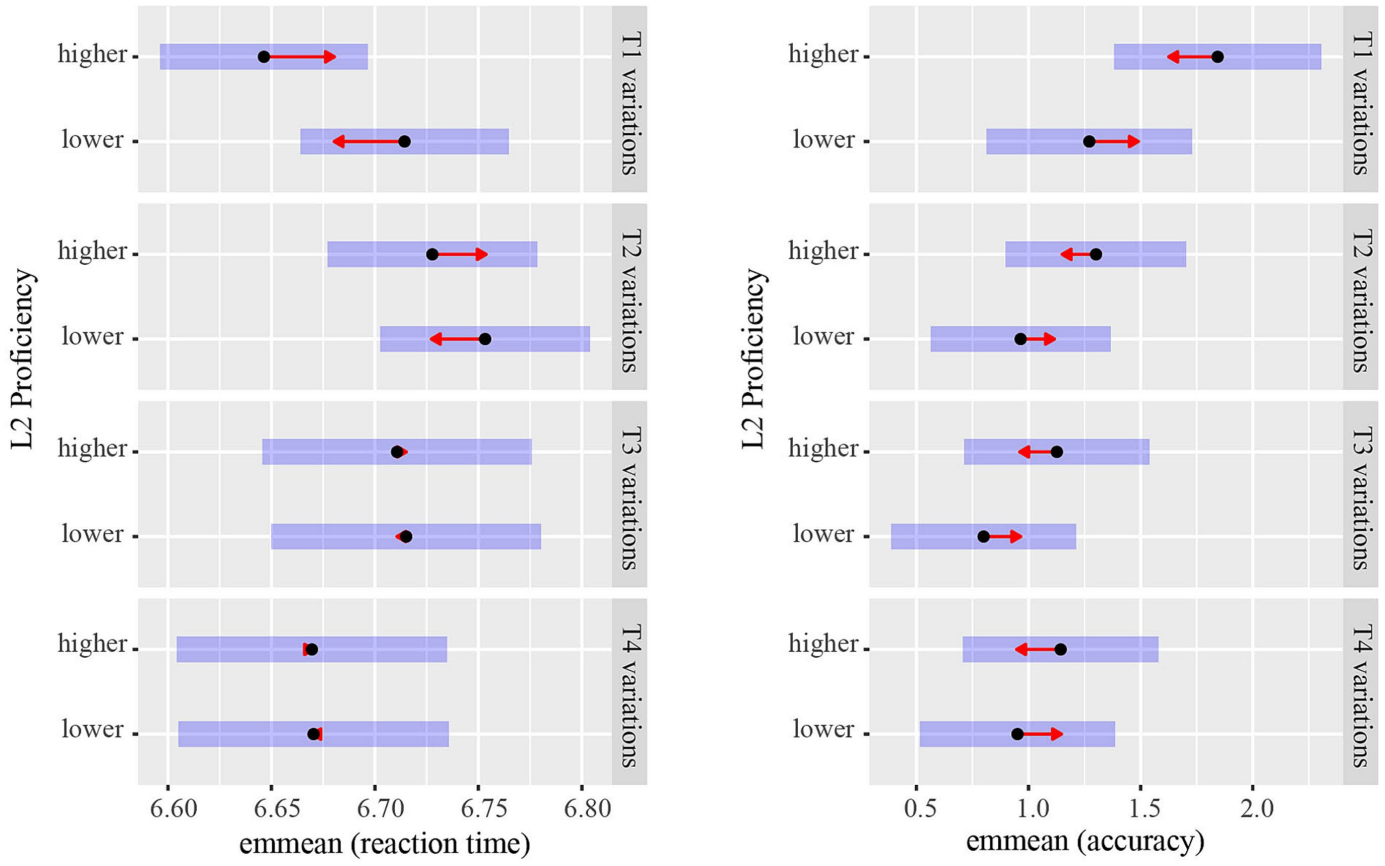
Group differences between bilinguals with higher and lower L2 proficiency in the perception of pitch height variations in T1, T2, T3, and T4. The figure shows estimated marginal means for reaction times and accuracy. Pairwise comparisons between proficiency groups were conducted within each condition. The higher proficiency group perceived pitch height variations in T1 faster, and those in T1, T2, and T3 more accurately, than the lower proficiency group. The extent to which arrows overlap reflects, as closely as possible, the significance of the comparison between the two estimates.

The results of the best model (with L2 proficiency as a categorical variable; see Table 4) showed that the effect of Condition was significant (T1 vs. T2 pitch changes: *β* = 0.06, *SE* = 0.02, *z* = 3.99, *p* < 0.001; T2 vs. T4 pitch changes: *β* = −0.07, *SE* = 0.02, *z* = −3.33, *p* = 0.001; T3 vs. T4 pitch changes: *β* = −0.04, *SE* = 0.02, *z* = −2.72, *p* = 0.007). In addition, the interaction between Condition and L2 proficiency was significant (*β* = 0.04, *SE* = 0.02, *z* = 2.01, *p =* 0.048), suggesting that the effect of L2 proficiency on pitch height changes in T1 was larger than that in T2 (see Figure 4). The other effects did not reach statistical significance (*ps* > 0.05).

**Table 4:** Mixed-effects model results for RTs in Experiment 2 with L2 proficiency as a categorical variable.

Fixed effects	*β*	*SE*	*t*	*p*
T1 as the reference level				
Intercept	6.68	0.02	352.48	<0.001***
Condition: T2	0.06	0.02	3.99	<0.001***
Condition: T3	0.03	0.02	1.63	0.107
Condition: T4	−0.01	0.02	−0.52	0.605
L2 proficiency	−0.07	0.03	−1.96	0.055
T2 × L2 proficiency	0.04	0.02	2.01	0.048*
T3 × L2 proficiency	0.06	0.03	1.88	0.065
T4 × L2 proficiency	0.07	0.03	1.98	0.052
T2 as the reference level				
Intercept	6.74	0.02	352.59	<0.001***
Condition: T3	−0.03	0.02	−1.35	0.181
Condition: T4	−0.07	0.02	−3.33	0.001**
L2 proficiency	−0.03	0.04	−0.73	0.469
T3 × L2 proficiency	0.02	0.03	0.61	0.547
T4 × L2 proficiency	0.02	0.04	0.68	0.500
T3 as the reference level				
Intercept	6.71	0.02	278.01	<0.001***
Condition: T4	−0.04	0.02	−2.72	0.007**
L2 proficiency	<0.01	0.05	−0.10	0.924
T4 × L2 proficiency	<0.01	0.02	0.16	0.876
T4 as the reference level				
Intercept	6.67	0.02	275.49	<0.001***
L2 proficiency	<0.01	0.05	−0.02	0.986

*p < 0.05; **p < 0.01; ***p < 0.001.

The *post hoc* analysis of Condition showed that perceiving pitch height changes in T1 was faster than those in T2 (*β* = −0.06, *SE* = 0.02, *z* = −3.99, *p* < 0.001, *d* = −0.23), and that perceiving pitch height changes in T4 was faster than those in T2 (*β* = −0.07, *SE* = 0.02, *z* = −3.33, *p* = 0.005, *d* = −0.27) and T3 (*β* = −0.04, *SE* = 0.02, *z* = −2.72, *p* = 0.033, *d* = −0.17). There were no significant differences (*ps* > 0.05) between the other conditions. The *post hoc* analysis of L2 proficiency showed no significant difference in the speed of perceiving pitch height changes of tones between the higher and lower level groups (*p* = 0.494). The *post hoc* analysis of the interaction between L2 proficiency and Condition (see Figure 4 and see text footnote 3) revealed that L2 proficiency influenced the perception of pitch height changes in T1 (*β* = 0.07, *SE* = 0.03, *z* = 1.96, *p* = 0.051, *d* = 0.26). Participants with higher L2 proficiency perceived pitch height changes in T1 faster than those with lower L2 proficiency. However, the perception of pitch height changes in T2 (*β* = 0.03, *SE* = 0.04, *z* = 0.73, *p* = 0.466, *d* = 0.10), T3 (*β <* 0.01, *SE* = 0.05, *z* = 0.10, *p* = 0.923, *d* = 0.02) and T4 (*β* < 0.01, *SE* = 0.05, *z* = 0.02, *p* = 0.986, *d* < 0.01) was not influenced by L2 proficiency.

Following the reviewer’s suggestion, we provide a brief report of the results from the model that included L2 proficiency as a continuous predictor (see text footnote 3). The effect of Condition was significant (T1 vs. T2 pitch changes: *p* < 0.001; T2 vs. T4 pitch changes: *p* = 0.001; T3 vs. T4 pitch changes: *p* = 0.007). The effect of L2 proficiency was also significant (on T1 pitch changes: *p* = 0.013). In addition, the interaction between Condition and L2 proficiency was significant (the effect of L2 proficiency on T1 vs. T2 pitch changes: *p* = 0.045). *post hoc* analyses of the interaction (see text footnote 3) showed that the simple effect of L2 proficiency on T1 pitch height variations was significant (*p* = 0.011), whereas the simple effects on T2, T3, and T4 pitch height variations were not significant (*ps* > 0.05).

#### 3.2.2 Accuracy

The results of the best model (with L2 proficiency as a categorical variable; see Table 5) showed significant effects of Condition (T1 vs. T3 pitch changes: *β* = −0.59, *SE* = 0.26, *z* = −2.30, *p* = 0.022; T1 vs. T4 pitch changes: *β* = −0.51, *SE* = 0.26, *z* = −1.97, *p* = 0.049). The effects of L2 proficiency were also significant (on T1 pitch changes: *β* = 0.57, *SE* = 0.22, *z* = 2.57, *p* = 0.010; on T2 pitch changes: *β* = 0.34, *SE* = 0.15, *z* = 2.18, *p* = 0.029). What’s more, the interaction between L2 proficiency and Condition was significant (*β* = −0.38, *SE* = 0.16, *z* = −2.37, *p* = 0.018), suggesting that the effect of L2 proficiency on T1 pitch changes was larger than that on T4 pitch changes (see Figure 4). The other effects did not reach statistical significance (*ps* > 0.05).

**Table 5:** Mixed-effects model results for ACC in Experiment 2 with L2 proficiency as a categorical variable.

Fixed effects	*β*	*SE*	*z*	*p*
T1 as the reference level				
Intercept	1.56	0.21	7.51	<0.001***
Condition: T2	−0.42	0.26	−1.63	0.102
Condition: T3	−0.59	0.26	−2.30	0.022*
Condition: T4	−0.51	0.26	−1.97	0.049*
L2 proficiency	0.57	0.22	2.57	0.010*
T2 × L2 proficiency	−0.24	0.16	−1.45	0.147
T3 × L2 proficiency	−0.25	0.16	−1.54	0.123
T4 × L2 proficiency	−0.38	0.16	−2.37	0.018*
T2 as the reference level				
Intercept	1.13	0.19	5.95	<0.001***
Condition: T3	−0.17	0.26	−0.66	0.509
Condition: T4	−0.09	0.26	−0.33	0.740
L2 proficiency	0.34	0.15	2.18	0.029*
T3 × L2 proficiency	−0.01	0.15	−0.06	0.950
T4 × L2 proficiency	−0.14	0.16	−0.92	0.360
T3 as the reference level				
Intercept	0.96	0.19	4.98	<0.001***
Condition: T4	0.08	0.25	0.33	0.740
L2 proficiency	0.33	0.17	1.93	0.053
T4 × L2 proficiency	−0.13	0.12	−1.13	0.257
T4 as the reference level				
Intercept	1.05	0.20	5.25	<0.001***
L2 proficiency	0.19	0.20	0.98	0.329

*p < 0.05; ***p < 0.001.

The *post hoc* analysis of Condition showed no significant differences (*ps* > 0.05) in the accuracy with which participants perceived T1, T2, T3, and T4 pitch changes. The *post hoc* analysis of L2 proficiency found that participants with higher L2 proficiency perceived pitch height changes in L1 tones more accurately than those with lower L2 proficiency (*β* = −0.36, *SE* = 0.16, *z* = −2.19, *p* = 0.029, *OR* = 0.70). The *post hoc* analysis of the interaction between L2 proficiency and Condition (see Figure 4 and see text footnote 3) indicated that the perception of pitch height changes in T1 (*β* = −0.57, *SE* = 0.22, *z* = −2.57, *p* = 0.010, *OR* = 0.56), T2 (*β* = −0.34, *SE* = 0.15, *z* = −2.18, *p* = 0.029, *OR* = 0.72) and T3 (*β* = −0.33, *SE* = 0.17, *z* = −1.93, *p* = 0.053, *OR* = 0.72) was influenced by L2 proficiency. That is, participants with higher L2 proficiency perceived pitch height changes in T1, T2 and T3 more accurately than those with lower L2 proficiency. However, the perception of pitch height changes in T4 was not influenced by L2 proficiency (*β* = −0.19, *SE* = 0.20, *z* = −0.98, *p* = 0.329, *OR* = 0.83).

The results of the optimal model with L2 proficiency entered as a continuous predictor (see text footnote 3) showed that the effect of Condition was significant (T1 vs. T3 pitch changes: *p* = 0.030). The effect of L2 proficiency was also significant (for T1 pitch changes: *p* = 0.015). In addition, the interaction between Condition and L2 proficiency was significant (the effect of L2 proficiency on T1 vs. T3 pitch changes: *p* = 0.004; the effect of L2 proficiency on T1 vs. T4 pitch changes: *p* < 0.001; the effect of L2 proficiency on T2 vs. T4 pitch changes: *p* = 0.018). Follow-up analyses of the interaction (see text footnote 3) showed that the simple effect of L2 proficiency on T1 pitch height variations was significant (*p* = 0.015), whereas the simple effects of L2 proficiency on T2, T3, and T4 pitch height variations were not significant (*ps* > 0.05).

### 3.3 Discussion

Experiment 2 examined the influence of L2 proficiency on the perception of pitch variations in L1 tones among Mandarin–English bilinguals. Two main findings emerged from the best-fitting models in which L2 proficiency was treated as a categorical variable. First, participants showed different sensitivity to pitch height variations across the four Mandarin tones. They were more sensitive to pitch height variations in T1 and T4, and less sensitive to pitch height variations in T2 and T3.^9^ Specifically, they perceived pitch height variations in T1 faster than in T2, and in T4 faster than in T2 and T3. Second, the interaction between Condition and L2 proficiency was significant, suggesting that the performance difference between participants with higher and lower L2 proficiency was larger for T1 pitch changes than for T2 and T4 pitch changes. Subsequent analyses showed that, compared to bilinguals with lower L2 proficiency, those with higher L2 proficiency perceived T1 pitch height variations more quickly, and were more accurate in perceiving pitch height variations in T1, T2, and T3. However, the perception of T4 pitch height variations was not influenced by L2 proficiency. These findings suggest that L2 proficiency differentially affects the perception of pitch height in the four Mandarin tones, positively influencing bilinguals’ perception of pitch height in T1, T2, and T3 but not in T4.

Similar to Experiment 1, the best-fitting model treating L2 proficiency as a continuous variable showed that some effects of interest were no longer significant in Experiment 2, possibly due to the limited sample size. Power simulations based on the current data and models with L2 proficiency treated as a continuous variable (see text footnote 3) indicated that, as the number of participants increased, the pattern of results was consistent with that obtained when L2 proficiency was treated as a categorical variable. Therefore, in the General Discussion, we primarily focus on the results in which L2 proficiency was analyzed as a categorical variable.

## 4 General discussion

In two experiments, we investigated how non-tonal L2 proficiency influenced the perception of tone and pitch contrasts in the tonal L1 among Mandarin–English bilinguals dominant in L1. The results suggest that the effects of L2 proficiency on the perception of tone pairs and pitch height variations in L1 tones are positive and selective. Specifically, for tone contrasts, bilinguals with higher L2 proficiency tended to be more sensitive to the T2-T4 contrast than those with lower L2 proficiency. However, the perception of the T2-T3 and T1-T3 contrasts was not influenced by L2 proficiency. Similarly, for pitch height contrasts, participants with higher L2 proficiency were more sensitive to pitch height variations in T1, T2, and T3 than those with lower L2 proficiency. However, the perception of pitch height variations in T4 was not influenced by L2 proficiency. In what follows, we elaborate on these findings, focusing on the effects of L2 proficiency on the perception of L1 tone and pitch contrasts.

### 4.1 The influence of L2 proficiency on the perception of L1 tone contrasts

Experiment 1 examined the influence of L2 proficiency on the perception of acoustically similar (T2-T3) and dissimilar (T2-T4 and T1-T3) tone contrasts in L1 among Mandarin–English bilinguals. The results suggest that L2 proficiency appears to selectively and positively affect the perception of certain tone contrasts. Specifically, L2 proficiency seemed to influence bilinguals’ perception of the T2-T4 contrast (marginally significant), but did not affect their perception of the T1-T3 or T2-T3 contrasts. Moreover, the possible influence of L2 proficiency on the perception of the T2-T4 contrast is positive, such that bilinguals with higher L2 proficiency tended to be more sensitive to the T2-T4 contrast (as reflected in numerically faster reaction times and higher accuracy) than those with lower L2 proficiency. The effect of L2 experience on L1 tone perception was also reported by Zinszer et al. (2015), who found an effect of L2 AoA on brain activation when bilinguals perceived within and across category tone pairs generated by T2 and T4.

The current results can be explained by cross-linguistic similarities in tone perception. English is a non-tonal language that does not use lexical tones, and its use of distinctive pitch at the word level is very limited compared with Mandarin. However, rising and falling tones are common in English intonation, and contrastive rising-falling pitch patterns can also be found in words (Chan and Leung, 2020; So and Best, 2010), as illustrated in the Introduction. Moreover, previous research shows that native speakers of English and Cantonese (another tonal language) exhibit similar performance in differentiating rising from falling tones (Chan and Leung, 2020), suggesting that native English speakers are sensitive to this contrast. The T2-T4 contrast in Mandarin reflects the difference between rising and falling tones, roughly corresponding to the rising and falling tones used in English intonation. Therefore, as L2 English proficiency increases, Mandarin–English bilinguals strengthen their perceptual ability for rising-falling pitch contrast and thus become more sensitive to the T2-T4 contrast in their L1.

### 4.2 The influence of L2 proficiency on the perception of L1 pitch contrasts

Experiment 2 investigated the influence of L2 proficiency on the perception of pitch height contrasts in the four L1 tones (T1, T2, T3 and T4) among L1-dominant Mandarin–English bilinguals. The results showed that L2 proficiency also positively and selectively influenced the perception of pitch height variations in L1 tones. Compared to bilinguals with lower L2 proficiency, those with higher L2 proficiency were more sensitive to pitch height variations in T1, T2, and T3. However, no effect was observed for pitch height variations in T4.

As mentioned in the Introduction, pitch height is a critical acoustic feature in both Mandarin and English. Furthermore, previous research has shown that native English listeners are more sensitive to pitch height than native Mandarin listeners (Jongman et al., 2017). The Mandarin–English bilinguals in our study had learned and used English for an average of about 12 years. Their ability to perceive pitch height may therefore have been strengthened during L2 English acquisition, making them more sensitive to pitch height variations in Mandarin tones. On the other hand, previous studies have found that native English speakers are less sensitive to T4/T4 variations in Mandarin than native speakers of tonal languages such as Mandarin and Cantonese (Jongman et al., 2017; So and Best, 2010). This suggests that phonological units at the lexical level, such as T4 (falling tone), may be non-salient in English, even though falling pitch is often used to express intonational decline at the sentence level in English. As a result, even with extensive exposure to English, Mandarin–English bilinguals may not show increased sensitivity to T4, which may in turn limit the transfer of perceptual sensitivity to pitch height to T4.

To sum up, the current study observed a positive effect of L2 proficiency not only on the perception of L1 tone contrasts, but also on the perception of L1 pitch height contrasts among L1-dominant Mandarin–English bilinguals. These findings suggest that increased non-tonal L2 proficiency enhances Mandarin–English bilinguals’ sensitivity to tone-related information in their L1. However, this positive effect is selective rather than universal. Specifically, tone/pitch features that are also present in English, or to which native English speakers are sensitive, such as rising-falling pitch contrasts and pitch height variations, are more likely to give rise to backward transfer (i.e., from L2 to L1). In contrast, Mandarin phonological units at the lexical level such as T4 are not salient in English, as evidenced by native English listeners being less sensitive to T4 variations than native Mandarin listeners (Jongman et al., 2017). Consequently, L2 experience may have limited influence on bilinguals’ perception of T4 variations in L1.

The findings of the current study support and extend the multi-competence theory to the domain of tone-related information processing. As stated before, the multi-competence theory focuses on the effects of L2 on L1 and categorizes them as positive, negative, and neutral (Cook, 2003; Cook and Wei, 2016). In addition, this theory proposes that negative effects typically occur in bilinguals whose L1 is not their dominant language, such as immigrants (Cook, 2003), which has been confirmed by prior research on tone processing in bilinguals (Quam and Creel, 2017). In contrast, the present study focused on L1-dominant Mandarin–English bilinguals, and observed a positive effect of L2 proficiency upon L1 tone and pitch processing. Moreover, the positive effect occurred selectively in phonological/acoustic features that are common to English and Mandarin. Thus, this study contributes to understanding how a non-tonal L2 may influence L1 tone processing. Note that the multi-competence theory is based primarily on findings from studies of non-tonal bilinguals and has mainly focused on L2 effects on higher level L1 processing, such as written expression and syntactic processing (Cook, 2003). The current study tested Mandarin–English bilinguals and further shows the positive effect of L2 experience on lower level L1 processing, namely the perception of tone-related information, and thereby extending the explanatory scope of the multi-competence theory.

Even though this study treated L2 proficiency as an independent variable and found that it affected L1 tone processing, the reasons behind these effects still need to be explored. We conducted further data analyses and ruled out general language-learning ability as a confounding factor. Specifically, we built mixed-effects models to examine the influence of L1 proficiency (self-rated) on the perception of tone pairs and pitch height contrasts. The results showed that neither the main effects of L1 proficiency nor its interactions with Condition reached statistical significance (*ps* > 0.05). Additionally, given that the tasks and stimuli used in the present study were fairly simple, we do not think that the current findings can be explained by higher cognitive abilities (e.g., memory or executive function). Moreover, the current results cannot be attributed to individual differences in overall tone discrimination, because the effects occurred only for phonological/acoustic features shared by English and Mandarin, not across all tone contrasts or in pitch height variations for all four tones. Nevertheless, this study cannot exclude the possibility that the positive effects observed in the T2-T4 contrast were due to higher proficiency individuals being better at learning the rising-falling pitch contrast, as noted by one reviewer. Finally, one limitation of the present study is that some key results reached only marginal significance, as noted above. This may be due to the limited sample size in the present study. This interpretation is supported by power simulations based on the current data, which indicate that increasing the sample size would allow the effects to reach 80% power or higher (for details, see text footnote 3). Future research should increase the sample size to avoid this limitation.

## 5 Conclusion

This study examined the effects of L2 proficiency on the perception of tone contrasts and pitch height variations in L1 tones among Mandarin–English bilinguals whose dominant language is Mandarin. The results suggest that L2 proficiency selectively and positively influenced the perception of tone pairs and pitch height contrasts. What’s more, the positive effects were observed for the phonological/acoustic features common to English and Mandarin. Specifically, compared to bilinguals with lower L2 proficiency, those with higher L2 proficiency tended to be more sensitive to the T2-T4 contrast and to pitch height variations in T1, T2, and T3. These findings suggest that proficiency in a non-tonal L2 enhances bilinguals’ sensitivity to tone-related information in their tonal L1, supporting and extending the multi-competence theory to the level of tone processing.

## Data Availability

The data, analysis code, results, and supplementary tables and figures for this study are available at: https://osf.io/q89sf/overview.

## Footnotes

1 The participants’ Mandarin was highly proficient, as schooling in China is conducted in Mandarin. In addition, previous research has shown that different dialect groups exhibit the same patterns in the categorical perception of Mandarin tones (Yu and Huang, 2019, *Applied Linguistics*—a journal published in China). Therefore, although participants in our study were likely exposed to other dialects, we did not collect information about such exposure.

2 In the subsequent statistical analysis, we included L2 AoA as a covariate in the optimal models. The results showed that L2 AoA did not significantly improve the model fit.

3
https://osf.io/q89sf/overview

4 Syllable duration was normalized in Praat using the following steps: (1) The target sound was selected; (2) *Modify → Modify times* was selected from the menu; and (3) *Scale times* was to be selected, with the value in the secondary input field set to 0.3.

5
http://www.fon.hum.uva.nl/praat/

6 Forward model comparisons for mixed-effects models are a strategy to optimize the model structure by adding fixed or random effects step by step, and are centered on assessing the significance of the model improvement through statistical indicators.

7 Power simulations were conducted using the *mixedpower* package in R. We simulated all fixed effects and their interactions for sample sizes ranging from 25 to 235 in steps of 10. The code for power simulations and the results are available on OSF (see text footnote 3).

8 The following steps were used to modify pitch in Praat: (1) The target sound was selected, and *Manipulate → To Manipulation* was chosen (retaining default settings), which created a new file; (2) The new file was selected, and *View & Edit* was clicked. All pitch points (i.e., the entire pitch contour) were selected; (3) *Pitch → Multiply pitch frequencies* was chosen, and a multiplication factor representing the desired pitch shift (e.g., 0.8 or 1.2) was entered; (4) Finally, *File → Publish resynthesis* was clicked to synthesize the modified sound.

9 This is likely because the fundamental frequency (F0) contours of T2 and T3 are more complex and quite similar (Jongman et al., 2006), making changes in their pitch height harder to perceive. The F0 contours of T2 and T3 both begin with a dip followed by a rise (So and Best, 2010). In contrast, the F0 contours of T1 and T4 are relatively simpler, with T1 being level and T4 being falling. Another possibility, as noted by the reviewer, is that T2 and T3 are realised with greater acoustic variation in Mandarin (e.g., tone sandhi). Moreover, studies on both Mandarin-speaking children and adults have found that they are more likely to confuse T2 and T3 than T1 and T4 in spoken word recognition and tone judgment tasks (Lee et al., 2009; Singh et al., 2016).
